# Conditionally unbiased and near unbiased estimation of the selected treatment mean for multistage drop-the-losers trials

**DOI:** 10.1002/bimj.201200245

**Published:** 2013-12-18

**Authors:** Jack Bowden, Ekkehard Glimm

**Affiliations:** 1 MRC Biostatistics UnitCambridge, CB2 OSR, UK; 2 Novartis Pharma AGCH-4002 Basel, Switzerland

**Keywords:** Bias-adjusted estimation, Drop-the-losers design, Treatment selection, UMVCUE

## Abstract

The two-stage drop-the-loser design provides a framework for selecting the most promising of *K* experimental treatments in stage one, in order to test it against a control in a confirmatory analysis at stage two. The multistage drop-the-losers design is both a natural extension of the original two-stage design, and a special case of the more general framework of Stallard & Friede (2008) (*Stat. Med*. **27**, 6209–6227). It may be a useful strategy if deselecting all but the best performing treatment after one interim analysis is thought to pose an unacceptable risk of dropping the truly best treatment. However, estimation has yet to be considered for this design. Building on the work of Cohen & Sackrowitz (1989) (*Stat. Prob. Lett*. **8**, 273–278), we derive unbiased and near-unbiased estimates in the multistage setting. Complications caused by the multistage selection process are shown to hinder a simple identification of the multistage uniform minimum variance conditionally unbiased estimate (UMVCUE); two separate but related estimators are therefore proposed, each containing some of the UMVCUEs theoretical characteristics. For a specific example of a three-stage drop-the-losers trial, we compare their performance against several alternative estimators in terms of bias, mean squared error, confidence interval width and coverage.

## 1 Introduction

The maximum likelihood estimate (MLE) of the treatment effect is often reported as standard at the end of a multistage trial. It is of course a precise and readily available estimator, but since it ignores the trial's sequential nature it is generally biased (Whitehead, 1986) and considerable research has been conducted into estimation methods that address this fact. Although many bias adjusted estimation procedures have been proposed, and unbiasedness is certainly not the only characteristic by which an estimator can be judged, the only way to achieve an efficient and “purely” unbiased estimate is to execute the following procedure: (i) identify an unbiased estimate based on part of the data—*Y* say, (ii) identify complete, sufficient statistics for the parameter in question—*Z* say, and (iii) employ the Rao-Blackwell improvement formula to obtain 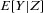
—the uniform minimum variance unbiased estimate (UMVUE).

There are two distinct applications of the Rao-Blackwell approach to estimation in multi-stage trials. The first approach, as pioneered by Emerson & Fleming (1990) and further clarified by Liu & Hall (1999), applies to a group sequential trial with one active treatment and one control arm that stops when conclusive evidence (for or against the efficacy of the treatment) is first observed. The stage at which the trial stops—*M* say—is a random variable. Given *M*, a sufficient statistic of the data, *Z*, and a truncation adaptive stopping rule (Liu & Hall, 1999) one calculates the expectation of the first stage data, *Y*_1_ say, given the pair 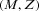 to obtain the truncation adaptable UMVUE. We refer to this approach as *unconditional* because, in a three-stage trial, for example, it produces an estimate of the treatment effect regardless of whether the trial stops at stage one, two, or three and it is therefore unbiased by definition when one averages across all possible realizations of the sequential trial.

However, in some circumstances one may feel it more appropriate to develop estimators that are UMVUE conditional on the occurrence of a particular subset of trial realizations. For example, in two-stage “drop-the-losers” trials, the best performing of *K* experimental treatments is selected after the first stage before being tested in isolation against a control group in a confirmatory analysis in stage two. The stage two estimate of the selected treatment, *Y*_2_ say, is unbiased, and Cohen & Sackrowitz (1989) obtain the UMVUE of the selected treatment, conditional on the order of the stage one treatment arm estimates, by calculating 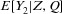, *Q* denoting the stage 1 order statistic condition. We call this an example of an UMVCUE—C for *conditional*. UMVCUEs have also been proposed for use more generally in two-stage trials evaluating a single treatment that allow early stopping for futility only. This is because a strong argument can be made that estimation of the treatment's effect is only important when the trial does in fact continue to the final stage; see Pepe et al. (2009), Koopmeiners et al. (2012), and Kimani et al. (2013) for recent examples.

The two-stage drop-the-losers' design has been the focus of much attention in the research literature. Sampson & Sill (2005) and Wu et al. (2010) consider hypothesis testing methodology for this design whereas Sill & Sampson (2007), Bowden & Glimm (2008), and Bebu et al. (2010) target point estimation. More generally, the vast majority of adaptive trial designs have also followed a two-stage strategy in the following sense: Design adaptations (such as subgroup selection or sample size adjustment) are made at the first interim analysis. The trial then proceeds under a fixed design (possibly with additional interim analyses) for its remaining duration. However, it is questionable whether a two-stage approach is always the best strategy. As an example, an adaptive trial was recently conducted into a treatment for maintaining lung function in patients with Coronary Obstructive Pulmonary disease (COPD) (Barnes et al., 2010). The trial aimed to use its first stage data to select the most promising doses of a new drug, before testing them against a placebo in a confirmatory analysis at stage two. In the event, two out of the four doses were selected for continuation to the second stage.

By selecting two doses (instead of one), the study decreased its chances of accidentally discarding a dose that would ultimately be successful. Nonetheless, evaluating two experimental treatments in the confirmatory analysis is more challenging than for one, since multiple testing corrections must be applied. Furthermore, if the best performing of the two experimental treatments ends up being recommended, then its estimate may subsequently be queried as “biased”. There may, therefore, have been an advantage in allowing selection of the single most promising treatment–dose to occur after several interim analyses. With this in mind, a clear multistage analog for the two-stage drop-the-losers design exists: rather than selecting the best of *K* treatments at a single interim analysis, selection could be achieved by dropping a predetermined number of treatments at each stage due to (relative) poor performance until only one remained. The downside of inserting additional interim analyses into any clinical trial is clearly an increased administrative burden. Yet, in the context of a drop-the-loser trial, doing so can markedly increase the probability of selecting the truly best treatment for a given number of patients, as is shown in Section 2012.

The multistage drop-the-loser design approach is actually special case of a more general design framework for testing multiple treatments proposed by Stallard & Friede (2008). In their paper, the decision to drop a treatment need not be dictated by a predetermined rule based on efficacy data but, if it is, the family wise error rate of the trial can be controlled in the strong sense. Stallard & Friede (2008) do not touch on the issue of estimation for their general design, although it is highlighted by them as an area for future methods development. In this paper we focus on estimation, and attempt to derive the UMVCUE for the specific drop-the-losers case. A different derivation to that of Cohen & Sackrowitz (1989) is used. It is perhaps less intuitive than the original, but generalizes to an arbitrary number of stages far more easily. Furthermore, allowing additional stages of selection requires an increasingly strong and unexpected form of conditioning to be employed. So in order to best elucidate the approach we start in Section 2012 by considering the extension to the three-stage case and the general *J*-stage formula is left as an appendix. In Section 4, we apply our estimation proposals to some specific three-stage trial examples, and compare its performance against several other estimation strategies. Interval estimation for the selected treatment is also considered. We discuss the issues raised and point to future research in Section 5.

## 2 The three-stage drop-losers design

Imagine a three-stage trial initially involving *K* experimental treatments and a control group. The purpose of the first two stages is to identify which treatment has the most beneficial true effect, as reflected via an appropriate outcome measure. Throughout this paper we will assume that higher values of the outcome are more desirable. At the end of stage one, 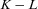 experimental treatments are dropped and then the best of the *L* remaining experimental treatments is selected at the end of stage two for confirmatory testing against the control group in stage three. We will refer to such a three stage design as a “*K*:*L*:1” trial. Let 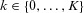 index the initial full set of treatments with 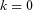 referring to the control group. Let 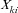 denote the response of the *i*th patient on treatment *k*, which is normally distributed with mean 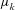 and variance 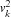. At stage *j* (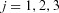), *n* subjects are randomized to each treatment arm still active in the trial and the experimental treatments are evaluated according to a test statistic of the form:

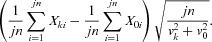
(1)Without loss of generality we will refer to the true mean of the selected treatment as μ_1_, the reasons for this subscript notation are given in due course. At the conclusion of the trial it would be natural to primarily focus on testing the null hypothesis 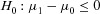. In this paper, we focus on the task of estimating the contrast 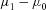. In the next subsection, we show empirically that whilst *K*:*L*:1 trials can provide more power to select the truly best treatment for confirmatory testing (compared to an analogous two-stage drop-the-losers trial) the MLE for μ_1_ can be substantially biased. Section 2.1 serves to merely illustrate the problem. Further details regarding the notation, as well as the design and analysis of drop-the-losers trials, are covered from Section 2.2 onwards.

### 2.1 Initial motivation

**Figure 1:**
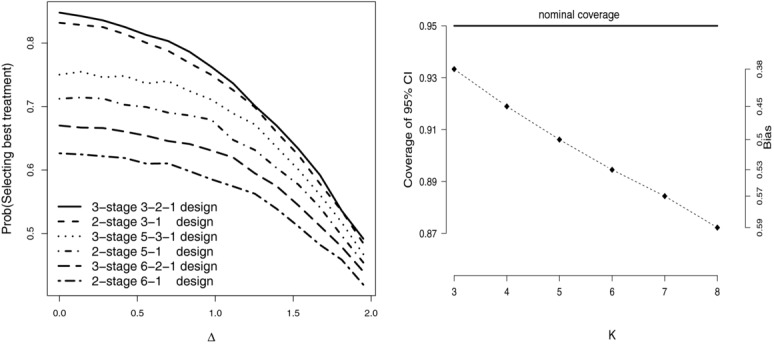
Left: power to select the truly best treatment for various two and three-stage drop-the-losers designs as a function of Δ. Right: bias and coverage of the MLE in a *K*:2:1 design, as a function of *K*.

Trial data are simulated under a 3:2:1 design. Each treatment arm at each stage is allotted *n* = 60 patients and the variance of each patient's individual treatment effect, 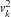, is 50. At the end of stage 2, 300 patients have been allocated to the experimental treatments. This is contrasted with a traditional two-stage “3:1” trial, where the same number of patients (100 per arm) are used to select the best performing treatment after stage one. We assume that the vector of true mean effects for the three treatments is (1, Δ, 2). Figure 1 shows the proportion of simulations for which the truly best treatment (with mean equal to 2) is selected, as Δ is varied between 0 and 2. Each point is the average of 50,000 simulations. The 3:2:1 design gives a marginally higher power of selecting the best treatment than the 3:1 design. The results of two further simulation scenarios are also shown in Fig. 1 (left), namely:

- A 5:3:1 trial (75 per arm per stage) compared with a 5:1 trial (120 per arm). True vector of treatment means (1, 1, 1, Δ, 2). 600 patients used to select best treatment in total.
- A 6:2:1 trial (75 per arm per stage) compared with a 6:1 trial (100 per arm). True vector of treatment means (1, 1, 1, 1, Δ, 2). 600 patients used to select best treatment in total.

For these scenarios, the difference in power between the two and three-stage designs is now much more pronounced, and the case for switching to a three-stage design is stronger. Increasing the sample size of the trial (i.e, reducing the variance of the estimates) will always increase the probability of selecting the best treatment (assuming that one is truly better than the others). In our example the treatment with mean effect 2 is always best. For increasing sample sizes, the power curves approach 1.

Note that, if equal numbers of patients were randomized to the control group as are to the experimental arms at each stage, the two and three-stage designs featured above would have different numbers of controls at different stages. However, since we have assumed that the trials always continue to the final stage, the control group data only impacts on the final stage analysis; it therefore does not affect the probability of selecting the best treatment.

Trial data is now simulated under a *K*:2:1 design for 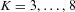 and with *n* = 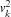 = 50 for all *k*. All treatments are assumed to have no (zero) effect. Figure 1 (right) plots the bias and coverage of the MLE for μ_1_ as a function of *K*. One can see that as the number of experimental treatments increases, the bias in the MLE increases and the coverage of its 95% confidence interval starts to fall well below its nominal level. In the context of a two-stage drop-the-losers trial, the bias in the MLE of the selected treatment is maximized when all treatments have the same effect (see Carreras & Brannath, 2013, for a proof). We conjecture that this is true under three-stage drop-the-losers selection as well. Despite the fact that Fig. 1 (right) most probably represents a worst case scenario in terms of the MLEs performance, there is certainly room for alternative estimation strategies to be developed.

### 2.2 Notation for the estimation problem

For simplicity we will now assume that the within treatment variance term 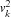 is constant across treatments, and is hence referred to as *v*^2^. This means that ranking the treatments via the test statistic in Eq. 2010 over the first two stages is equivalent to ranking by the values of the experimental treatment terms only. That is, the cumulative control group data at stage *j*, 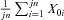, and the common square root term 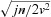 can be ignored, leaving the experimental treatment MLEs to be directly compared head-to-head.

Let 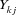 (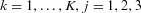) represent the estimate for the mean effect of treatment *k* using only those subjects recruited at stage *j*. In order to add more flexibility, let the originally fixed number of subjects per-arm per-stage, *n*, now equal 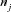, so this number can vary across stages if required (as in Bowden & Glimm (2008)). Letting 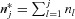, and 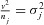, then:

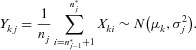
We base all subsequent mathematical derivation on the 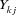 random variables (or transformations of them), leaving the individual patient data 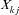 notation behind. Let 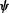 represent the vector (ψ_1_, …,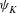) where 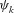 is the ranking of treatment *k* using our design framework. Denote the event: 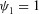, 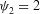, …, 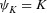 by the letter *Q*. It is useful to label the 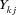
s so that event *Q* is satisfied. That is, so that 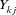 refers to the treatment that is ultimately ranked as *k*th best at the end of the trial. However, it is important not to confuse or equate this convenient labeling with explicitly conditioning on event *Q*—this is done in Sections 3 and 3.1.

**Figure 2:**
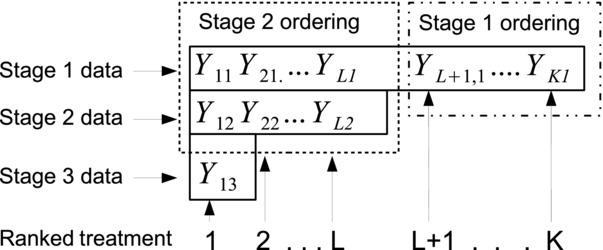
Schematic diagram showing the selection process of the three stage trial.

At the end of stage 1, the top *L* treatments are kept and the remainder are dropped. So, 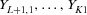 (which are identical to the stage one MLEs) satisfy:

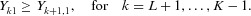
(2)This enables 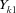 and 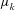 to be associated with treatments 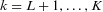 after stage one. At stage two, the remaining *L* treatments are ranked according to their cumulative MLEs. So, assume that the remaining stage one statistics 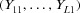 and 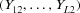 satisfy: 

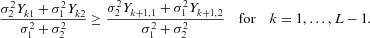
(3)This enables 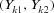 and 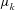 to be associated with treatments 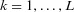 at stage 2. A schematic diagram of this selection process is shown in Fig. 2. Ultimately, the selected experimental treatment is therefore associated with stage-wise statistics 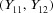 and additionally a *Y*_13_ ∼ N(μ_1_,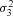), the sole experimental treatment tested at stage 3. Note that although 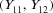 fulfills the inequalities in Eqs. (2) and (3), this does not directly imply that 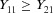 or 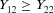. Note further that although the truly best treatment has the highest chance of being selected, μ_1_ is not equivalent to max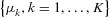.

At the third and final stage of the trial, we seek an efficient unbiased estimate of μ_1_ − μ_0_, where μ_0_ represents the mean parameter of the control group. As previously mentioned, the control group always progresses to the final stage of the trial, so μ_0_ can be trivially and unbiasedly estimated using all of the relevant data via its MLE. By contrast, the parameter μ_1_ is much more elusive; at the trial outset it is a discrete random variable with *K* possible values and, conditional on *Q*, it is *not* unbiasedly estimated by its final stage three MLE:

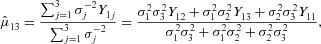
(4)since *Y*_11_ and *Y*_12_ are conditionally biased with respect to μ_1_. We therefore focus on bias-adjusted estimation of μ_1_ for the remainder of the paper.

## 3 Unbiased and bias-adjusted estimation of μ_1_

Let 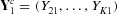 and 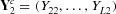 represent the vector of mutually independent unselected treatment estimates at stages one and two. Further, let **Y**_1_ and **Y**_2_ represent the complete set of normally distributed statistics at stage one and two so that, for example, 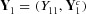. The joint distribution, *f*(.), of the complete data 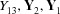:

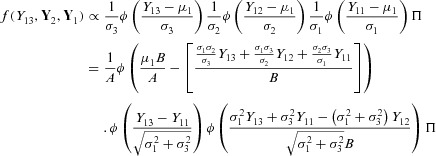
(5)where 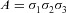, 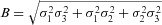 and Π equals all terms involving 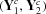. The trio 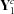, 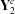 and 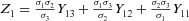 are therefore complete sufficient statistics for all mean parameters unconditionally. We note that the conditional density 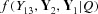 is essentially the same as Eq. (5), except that its support is restricted by *Q*. It must therefore be scaled up by a factor representing the probability of *Q*, in order to integrate to one over the restricted space.

In the spirit of Cohen & Sackrowitz (1989) who investigated two-stage UMVCUEs, a three-stage UMVCUE for μ_1_ would be a Rao-Blackwellization of the unbiased final stage estimate *Y*_13_ conditional on the complete sufficient statistic 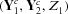 and under *Q*. We now clarify precisely how *Y*_13_ is restricted in this setting. At stage one, we can state from Eq. 2008 that (a): 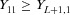 and from Eq. (3) (by removing a common term 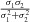) that:

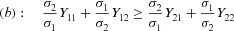
The set of *Y*_13_ values that satisfy conditions (a) and (b) is the sampling distribution of 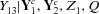. From the definition of *Z*_1_, condition (b) implies that:

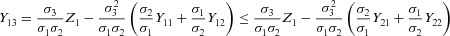
and condition (a) implies that:

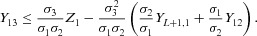
Therefore, 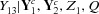 must be less than

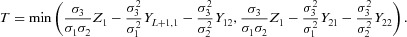
Since *T* depends explicitly on the value of *Y*_12_ (as well as implicitly through *Z*_1_) it is natural to condition on (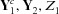) rather than on (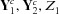) in order to calculate 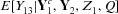. We will refer to this as the “RB1” estimator, and denote it by the symbol 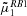.

Lehmann & Scheffe (1950) have shown that a sufficient and complete statistic is also minimally sufficient (the converse is not true). Therefore, since (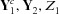) is sufficient but not minimal, it cannot be complete. Thus, the RB1 estimator will be unbiased and have a smaller variance than *Y*_13_ by the Rao–Blackwell theorem, but it is not UMVCU. We now calculate 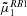, returning to a discussion of what properties can and can not be claimed by it in Section 3.3.

### 3.1 Deriving the RB1 estimator

We will derive the RB1 estimator in the following manner. We start by transforming:

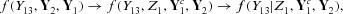
then show that *Y*_13_ is independent of (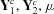) given (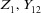), and finish by going from:

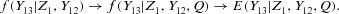
Let the vector 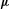 and diagonal matrix Σ be the mean and variance of the 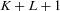 variables 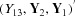 written as 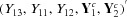. They follow the ordering described in Section 2.2 and Fig. 2 so that, if 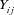 is the *l*th variable, the *l*-th entry of 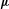 is 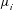 and the (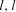)-th element of Σ is 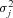. Using a standard result (e.g., Srivastava, 2002, Theorem 2.5.1), 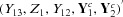 follows the (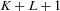)-dimensional multivariate normal (MVN) distribution: 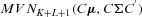, where

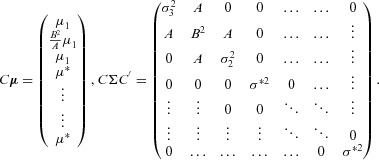
For convenience we have labeled the mean and variance parameters of 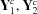 using the generic symbols 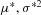, not because they are all equal, but because they are equally irrelevant to subsequent development. Next, we apply another well known theorem on conditional multivariate normal distributions (e.g., Srivastava, 2002, Theorem 2.5.5). Let

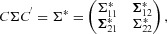
where 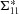
= 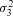 and 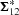 is equal to the 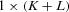 vector 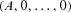 = 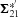. The inverse of the remaining 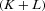 × 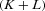 submatrix, 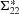, equals

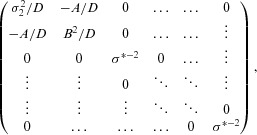
where 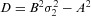. Defining 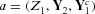, and 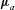 = 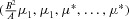, we can write the conditional distribution 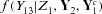 as 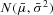 where:

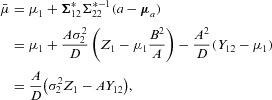
and 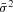 = 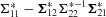 = 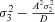. The conditional density of *Y*_13_ given *Z*_1_ and *Y*_12_ does not depend on 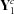, 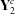 or μ. We therefore drop irrelevant terms by writing it as 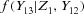. Only now do we condition on event Q, which acts to restrict 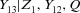 to be 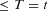
—the value of *T* being fixed by the observed values of 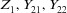 and 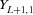 (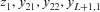). This yields the density

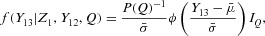
where 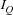 is the indicator function for event *Q* and

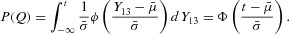
Taking the expectation of 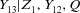 yields

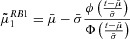
(6)

### 3.2 Deriving the RB2 estimator

The 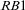 estimator in 2008 does not look like a direct analog of the two-stage UMVCUE of Bowden and Glimm (2008)—a correction to the two-stage MLE—because of the extra conditioning on *Y*_12_. One way of avoiding this extra conditioning would be to calculate 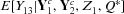, for a 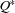 that did *not* depend on *Y*_12_. This can be achieved by defining the condition 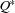 simply as the portion of *Q* coming from the stage two selection rule (b). We can then state that 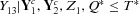 where:

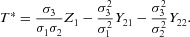
The quantity 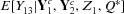 can then be obtained following the procedure as in Section 3.1, to yield an alternative estimator for μ_1_, which we will denote as 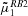 and refer to as the “RB2” estimator. It has the same form as 2008 but with *t* replaced by the observed value 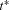 of 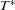, with 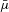 equal to the MLE 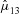 and with 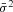 equal to 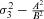.

### 3.3 Summarizing the RB1 and RB2 estimators

The RB2 estimator is the expected value of *Y*_13_ given the complete sufficient statistic and under condition 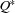. So, if 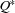 truly represented the condition restricting 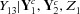 under drop-the-losers selection, then 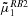 would be the UMVUE given 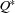. However, no such claim can be made because 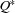 is not the correct condition, it is *Q*, so *E* [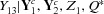] is conditionally biased with respect to μ_1_. Likewise, the RB1 estimator can not claim to be the UMVUE, conditional on *Q*, because it does not condition on a minimal sufficient (and hence complete) statistic.

One could argue, however, that if we simply condition on *Y*_12_ and *Q* first, then (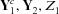) is a minimal sufficient statistic and therefore the RB1 estimator is a UMVCUE of sorts. This might be perceived as simply a semantic trick, and it is clear that conditioning on *Y*_12_ is not as natural as conditioning on *Q* alone. For this reason we will refer to 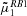 and 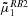 as simply Rao-Blackwellized estimators.

#### 3.3.1 A general formula for the RB1 estimator

In the Appendix, we derive the 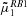 estimator for a *J*-stage drop-the-losers trial. This estimator will yield a more efficient, unbiased estimate of μ_1_ than using the stage *J* data alone. For the *J*-stage case, one is forced to condition on *Z*_1_ and 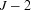 additional variables corresponding to the individual treatment effect estimates of the ultimately selected treatment at stages 2 to 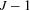. Although the precise values of 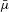, 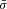 and *t* change, the *J*-stage estimator is identical in form to Eq. (6). Since it only ever requires the evaluation of a standard normal density and distribution function, it remains trivial to evaluate whatever the value of *J*.

### 3.4 Strength of unbiasedness of the RB1 estimator

Since μ_1_ is a random variable, it is convenient to consider the extended definition of bias due to Posch et al. (2005). That is, for a generic estimator 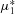 of μ_1_, the bias is given by:

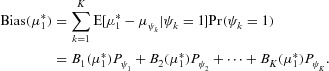
(7)Here 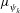 refers to the true mean of treatment *k*, the *k*-th element of μ. 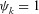 is the condition that treatment *k* is selected under the design and 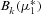 represents the bias of estimator 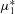 conditional on treatment *k* being selected. Although 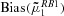 = 0, it actually fulfills a stronger form of unbiasedness, namely that 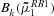 = 0 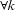.

### 3.5 An alternative near-unbiased estimator

In Section 4, we show the price paid by the RB1 estimator for unbiasedness is a substantial increase in mean squared error (MSE). Part of the motivation for developing the RB2 estimator was to see if it could trade off small amounts of bias for a MSE reduction. An additional bias-adjusted (but not unbiased) estimator is now considered. Bebu et al. (2010) proposed a likelihood based procedure for obtaining a bias corrected MLE (which we will refer to as the BC-MLE) and confidence intervals for the selected treatment in a two-stage drop-the-losers trial. We now adapt their general approach to the specific example of a 3:2:1 drop-the-losers design setting in order to complement the simulations in Section 4. Extensions to the general three stage case are obvious, but when more treatment arms are added the computational effort in obtaining the BC-MLE increases markedly.

Assuming that the variance of all stage-wise statistics are known, the log-likelihood of the parameter vector 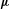 conditional on Q:ψ=(1,2,3) is proportional to

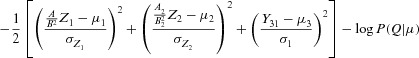
(8)

Here, 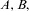 and *Z*_1_ are as defined in Section 3 after Eq. (5), 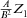 being equal to the selected treatment's MLE with variance 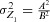. 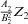 is defined here as the MLE of the second best treatment at stage 2, 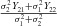, with mean μ_2_ and variance 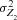 = 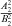 = 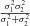. *Y*_31_ is the sole statistic on the treatment that ranked last at stage one, with mean μ_3_ and variance 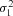. The penalizing term 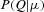 represents the probability of event *Q* given μ. This is equivalent to the probability that all three elements of the multivariate density:

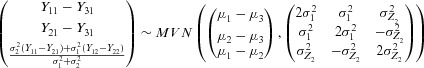
are positive, and it can be approximated to a high degree of accuracy in R using the pmvnorm() function (Genz & Bretz, 2009). The conditional log-likelihood 2010 can then be maximized to yield joint estimates for μ_1_, μ_2_, and μ_3_.

## 4 Simulation studies

We now conduct a simulation study to compare the performance of the MLE, RB1, RB2, BC-MLE, and stage three estimator *Y*_13_ in estimating μ_1_ under drop-the-losers selection. Various parameter constellations for the true treatment means and the stage-wise variances are considered. We note that in a real trial setting the quantity of interest would be the treatment versus control group comparison 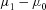, but for reasons already discussed we can ignore estimation of μ_0_. By averaging over the results of all simulations, we obtain a Monte–Carlo estimate for the estimators' bias as given in Eq. (7). By summing the estimators' squared errors across all simulations, we can approximate their MSE, when defined analogously to Eq. (7) as well.

### 4.1 Point estimation for a 3:2:1 trial

**Figure 3:**
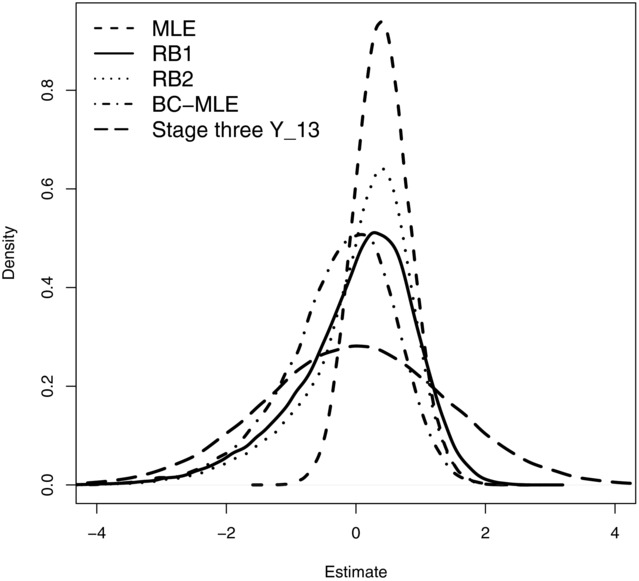
Distribution of the 5 estimators' estimates for 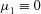 from a three-stage 3:2:1 drop-the-losers design, given stage-wise variances 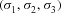 = 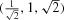. Each distribution is based on 100,000 simulations.

In a simple initial simulation, all three treatments are assumed to have a true mean effect of 0, so that μ_1_ ≡ 0. The number of patients recruited to each remaining treatment arm at each stage (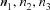) is 100, 50, and 25, respectively. *v*^2^ = 50 so that 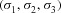 = 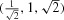. Figure 3 shows the distribution of 100,000 realizations of the MLE, RB1 estimator, RB2 estimator, BC-MLE, and *Y*_13_. From this simulation their empirical biases and MSEs were (0.41, 0.00, 0.04, −0.26, 0.00) and (0.35, 0.85, 0.69, 0.80, 2.00), respectively. *Y*_13_ is unbiased but inefficient and at the other extreme the MLE is efficient but biased. The RB1 estimator is unbiased and has a substantially lower MSE than *Y*_13_ because it utilizes data from the first two stages. Incorrect conditioning induces a small amount of bias into the RB2 estimator but is accompanied by a substantial decrease in MSE. The BC-MLE reduces the magnitude of the bias in the MLE but is shown to overcorrect. Its modal value is, however, close to the true value of 0.

In Table 1, we show the bias and MSE of the estimators for this choice of parameter values and three additional true parameter constellations. Trial data were simulated with 50 patients per-arm per-stage so that 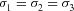 = 1. The same general pattern is observed across all scenarios, except the magnitudes of the biases and MSEs change.

**Table 1:** Bias and MSE of the various estimands over the four scenarios (50,000 simulations per scenario). In each case 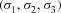 = (1, 1,1)

Parameter values	MLE	RB1	RB2	BC-MLE	Stage three *Y*_13_
	Bias				
(0, 0, 0)	0.377	−0.005	0.038	−0.142	−0.004
(0, 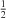 , 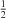 )	0.357	0.007	0.043	−0.132	0.007
(0, 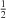 , 1)	0.310	0.004	0.041	−0.122	0.012
(0, 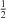 , 2)	0.111	−0.005	0.010	−0.092	0.012
	MSE				
(0, 0, 0)	0.388	0.690	0.563	0.579	0.982
(0, 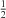 , 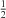 )	0.384	0.686	0.550	0.570	1.002
(0, 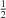 , 1)	0.372	0.670	0.524	0.553	1.017
(0, 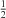 , 2)	0.347	0.587	0.413	0.471	1.019

### 4.2 Interval estimation for a 3:2:1 trial

It is possible to derive an expression for the variance of the RB1 estimator using the delta method, as, for example, Koopmeiners et al. (2012) do in the context of a single arm two-stage trial with a binary endpoint. However, from Fig. 3 we see that, in the context of a three-stage drop-the-losers trial, the distribution of 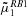 is highly skewed. Therefore, even if we were to derive analogous expressions for the variance of these quantities, it would not appear sensible to use them to furnish symmetric confidence intervals around the point estimate. For this reason, we adapt the nonparametric bootstrap procedure—originally proposed by Pepe et al. (2009) for a single arm two-stage trial—to the three-stage drop the loser setting. Specifically, we perform the following resampling schema to trial data 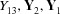:

1. Produce bootstrap sample of first stage data, with mean 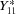.
2. If 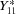 is ≥ original observed value 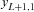:
  - Produce bootstrap samples of second stage data, with mean 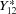.
  - If stage two MLE 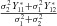 is ≥ original observed value 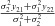:
    - Produce bootstrap samples of third stage data, with mean 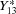.
    - Calculate the RB1 estimator 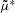 from Eq. (6) given 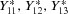, and original observed value *t*.

This should be repeated until a large enough collection of 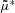
s have been obtained to accurately assess its sampling distribution. Empirical quantiles of this distribution can then be read-off to give confidence intervals for 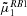. Upon implementing this procedure, it is no extra effort to additionally calculate bootstrapped confidence intervals for the RB2 estimator at the same time.

Confidence intervals for the BC-MLE of μ_1_ are obtained by the profile likelihood approach described in Bebu et al. (2010). That is, we calculate the statistic 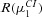 as twice the difference between the log-likelihood evaluated at the joint BC-MLEs for 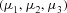 and at the BC-MLEs for 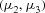 given the constraint 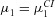. A (1 − α) level confidence interval for μ_1_ is then the set of values for which 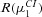 ≤ 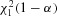.

Table 2 shows, for 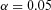, the average confidence interval width and the resulting coverage of the BC-MLE, RB1, and RB2 estimators for the four scenarios already introduced and with 50 patients per treatment arm per stage as before. Within each simulation, confidence intervals and coverage were assessed with respect to the true (fixed) value of μ_1_, and then averaged across simulations. Each bootstrapped confidence interval calculated was based on 1000 simulated values of 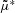. The overall figures are based on only 10,000 simulations—obtaining a confidence interval for the BC-MLE using the profile likelihood method required substantially more computational effort than the bootstrap procedure, and so this was the limiting factor.

**Table 2:** Coverage and mean confidence interval width for the BC-MLE, RB1, and RB2 estimators. (10,000 simulations per scenario). In each case 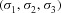 = (1, 1, 1)

	RB1		BC-MLE		RB2	
Parameter values	Cov 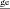	CI width	Cov 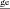	CI width	Cov 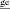	CI width
(0, 0, 0)	0.963	3.31	0.949	2.92	0.951	2.91
(0, 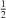 , 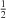 )	0.958	3.27	0.950	2.90	0.949	2.87
(0, 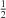 , 1)	0.954	3.20	0.948	2.83	0.946	2.80
(0, 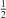 , 2)	0.956	2.96	0.945	2.57	0.956	2.49

Both the bootstrap and profile likelihood approaches appear to provide confidence intervals with nominal coverage. The RB1 estimate's confidence interval width is by far the widest. The smallest width is obtained from the RB2 estimator, but it is only marginally smaller than that of the BC-MLE.

### 4.3 Further results for *K*:2:1 trials

**Figure 4:**
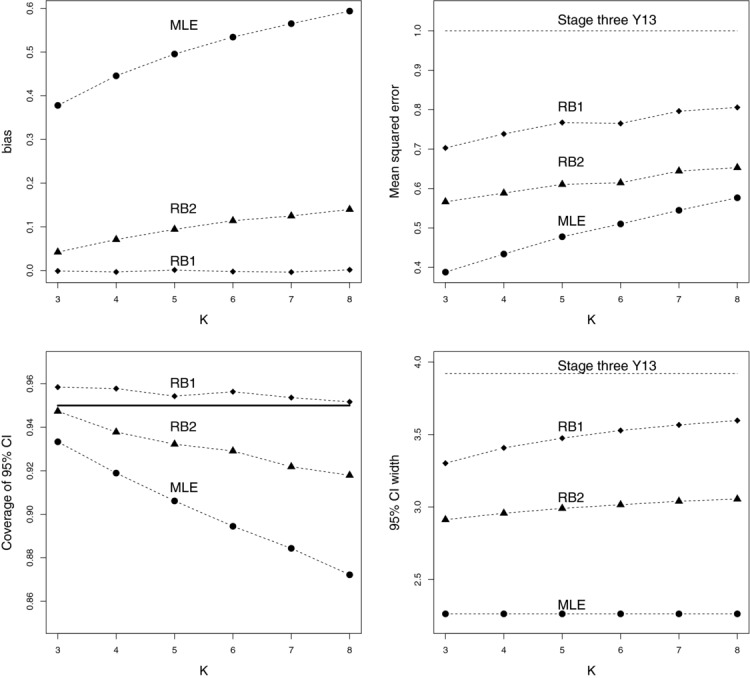
Bias, MSE, coverage (w.r.t. 95% confidence intervals), and average 95% confidence interval width of the MLE, RB1, and RB2 estimators as a function of *k*, for 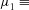 0, σ_1_=σ_2_=σ_3_=1. The stage three estimate *Y*_13_ is also shown where informative.

Trial data is now simulated under a *K*:2:1 design with all treatment means equal to 0 (so that 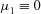), as in Section 2.1. Figure 4 (top-left to bottom-right) plots the bias, MSE, coverage and confidence interval width of the MLE, RB1, and RB2 estimators as a function of *K*. The properties of the stage three estimator *Y*_13_ are shown where informative (it is unbiased, with a known variance, so a simple symmetric confidence interval around it will achieve its nominal coverage). Each point is based on 50,000 simulations. The BC-MLE is not evaluated as it becomes too computationally demanding. However, there is no reason to suspect that its performance significantly worsens as more treatment arms are added. Standard symmetric confidence intervals are used for the MLE, assuming a normal distribution, and ignoring selection. While this could be termed a “standard” analysis, there is of course no reason to believe that this confidence interval will achieve its nominal coverage probability. As *K* increases, the bias of RB2 estimator increases but stays at modest levels compared to the MLE. The RB1 estimator and *Y*_13_ are unbiased. The MSE of the MLE is substantially lower than the other two estimators when *K*=3, but rises more quickly than the other two as *K* increases. The MSE of *Y*_13_ is 1, by definition.

The coverage of the RB1 estimator's bootstrapped 95% confidence interval stays relatively constant over the range of *K*, but is always slightly conservative. The RB2 estimator's coverage starts to worsen as *K* increases, but not to the same extent as the MLE. Table 3 shows, for these three estimators, the proportion of times that their 95% confidence intervals are above or below the true value of μ_1_. In each case, being above the true value is far more likely. This can be understood by the fact that all three are more likely to over-estimate than under-estimate the true effect. For example, in the simulations shown in Fig. 3 the MLE, RB1, and RB2 estimators overestimate μ_1_ 83%, 57%, and 61% of the time, respectively. Of course, in the case of the RB1 estimator, this tendency to overestimate is perfectly cancelled out by less frequent, but larger, underestimation so that 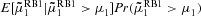 + 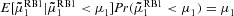. While the above/below ratio of the RB1 estimator stays fairly constant (around 10:1), the RB2 and MLE above/below ratio increases rapidly with increasing *K*. This reflects their increasing positive bias.

**Table 3:** Proportion of times the 95% confidence interval for the MLE, RB1, and RB2 estimators are above or below μ_1_

	RB1		RB2		MLE	
K	% Above μ_1_	% Below μ_1_	% Above μ_1_	% Below μ_1_	% Above μ_1_	% Below μ_1_
3	3.9	0.48	5.3	0.130	6.4	0.084
4	4.0	0.34	6.2	0.052	7.9	0.044
5	4.2	0.32	6.9	0.044	9.6	0.012
6	4.1	0.31	7.1	0.044	10.0	0.016
7	4.3	0.30	7.7	0.024	12.0	0.016
8	4.3	0.32	8.0	0.032	13.0	0.012

The MLEs average confidence interval width (a constant value of 3.92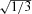) is far lower than the other two estimators—the RB1 estimator's confidence interval is on average 60% wider than the MLEs when *K* = 8, but the MLE suffers from suboptimal coverage as a result. The confidence interval width of *Y*_13_ is a constant value of 3.92, which gives an idea as to the additional gain in using the RB1 estimator, if unbiased estimation is required.

## 5 Discussion

In this paper, we have explored the issue of estimation for a multistage analog of the two-stage drop-the-loser design. Our main focus was to generalize the work of Cohen & Sackrowitz (1989) to enable efficient unbiased estimation of the selected treatment. In this regard, we can only claim to have been partially successful. The RB1 estimator is unbiased and has a lower variance than the final-stage estimator, but it is derived using an additional condition that is needed to overcome technical difficulties. Further work may reveal that these conditions can be relaxed to yield more efficient unbiased estimators. Perhaps unsurprisingly, the RB1 estimator was shown to have a large MSE, due to its unbiasedness. The RB2 estimator was derived as an alternative; its less stringent conditioning (on the minimal sufficient statistic) resulted in an estimator with a small amount of bias but a greatly reduced MSE in the context of a 3:2:1 trial. However, its performance (in particular the coverage of its bootstrapped confidence intervals) worsened for K:2:1 trials as *K* increased.

Our derivation of the RB1 and RB2 estimators assumes that the within treatment arm variances (the 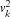
s) and the number patients randomized to each treatment arm at stage *j* (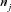) are equal across treatments. This meant that ranking via the test statistic in Eq. (1) is equivalent to ranking by the mutually independent experimental treatment MLEs at each stage. Indeed, the independence property gained by ignoring the common control group data in the selection process is key to the proof. If these two conditions are not met then ranking via test statistic and MLE will not be equivalent, so the RB1 and RB2 estimators as stated here will be invalid. Our development also assumes that the 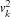 terms are known and a very different approach would be required if they were assumed unknown (Cohen & Sackrowitz, 1989). The BC-MLE approach of Bebu et al. (2010) is, in contrast, much better suited to the unknown variance case, since they can simply be included as additional parameters in their conditional likelihood.

The multistage drop-the-losers design assumes that the trial *always* proceeds to the final stage. Whilst this could be criticized as nonsensical and inefficient when strong evidence exists to stop the trial early, it gives the trial a fixed sample size that may make it attractive to practitioners and funding bodies alike (Kairalla et al., 2012). One may, however, wish to augment this design with an efficacy/futility stopping rule, as do Stallard & Friede (2008) and Wu et al. (2010). Our approach can easily be adapted to yield a RB1 or RB2 estimator in this context, by recalculating (in the three stage case) exactly how the sampling space of *Y*_13_ was restricted conditional on 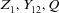 *and* given the trial made it to the final stage. However, from an estimation perspective, conditioning on reaching the final stage only really makes sense when the trial can stop early for futility and not efficacy (Pepe et al., 2009). Moreover, in this case whilst estimation of μ_1_ is still trivial, unbiased estimation of the treatment control comparison 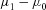 does not immediately follow, because the control group data must itself be corrected for some selection bias induced by the stopping rules. This extra complication has, however, been successfully addressed in recent work by Kimani et al. (2013).

Further research is needed to explore and understand how best to design and analyze *J*-stage drop-the-losers trials from an operational planning and hypothesis testing perspective. For example, how to calculate the critical value for testing the selected treatment against the control at the final stage, whether it is possible to control the type I error rate of this test in the strong sense; finding multistage designs (e.g., the *K* and *L* in the three stage context) that are optimal—in terms of maximal power and minimal size. Some preliminary work on this subject can be found in a technical report (Wason & Bowden, 2012) available at https://sites.google.com/site/jmswason/supplementary-material. Software (in the form of R code) to reproduce our results can be found in the supplementary material accompanying this paper.

## Appendix

### The RB1 estimator for a J-stage drop-the-losers trial

In this section, we introduce a more general notation. Let  represent the number of experimental treatment arms active in the trial at stage *j*, for . Clearly  and . Define  to be the set of all treatment arms in the trial at stage *j*, so that:

Let  represent the effect estimate of treatment *u* at stage *j* for . Let  be the standard deviation of the estimates at stage *j*. Further define:

Let  represent the *J* effect estimates associated with the (ultimately) selected treatment with true mean effect μ_1_. Let  represent the vector of the unselected treatment effect estimates at stage *j*. The vector of all treatment estimates across all stages can be written as  and has length *N* = . The MLE of μ_1_ at stage *j*, , is equal to

(A1)

Define  and rewrite  in terms of *Z*_1_ and  as

As for the three-stage example in Section 2012, in the first  stages of a *J*-stage trial, we sequentially rank the treatment arms by the order of their cumulative MLEs, defined for each treatment remaining in the trial at stage *j* as in Eq. (A1). The *j*-th stage imposes the restriction:

Given *Z*_1_, when  satisfies all of  selection conditions required by event *Q*, it is restricted to be less than or equal to

(A2)

where  represents the treatment effect estimate from stage *u* associated with the ()-th largest cumulative MLE at stage *j* and the second summation is only defined and evaluated if . For example, in Section 2012 we set *J*=3 and conditions (a) and (b) on *Y*_13_ correspond to setting *j* equal to 1 and 2 in formula (A2) respectively. This suggests that when conditioning  on Q, we additionally need to condition on  when calculating the RB1 estimator. Let *Y*_11_, **Y**_1_,  and  represent the complete data from the *J*-stage trial. Following the previous development in Section 3, we shall transform the multivariate normal densities as follows:

Upon demonstrating that  is independent of  given  we finish by going from

The density  is  where:

Letting  we define:

where = ,  is equal to the  vector  =  and  is the remaining  ×  matrix. The conditional distribution  is , where:

for ,  equal to mean parameter vector of *a* and . Using block-wise inversion techniques (e.g. Srivastava, 2002, Corollary A.5.2)  can be expressed as

where *M*_1_ is the scaler ,  and the values of *M*_3_ and *M*_4_ are unimportant. We now see that

and  = . Noting that this distribution does not depend on  or  we write  as

and  equals

(A3)

The J-stage RB2 estimator could be derived as in Section 3.2, by ignoring the first  selection steps that depend on . However, the bias of the RB2 estimator is likely to be increasing as a function of *J*.

## Notes

As a service to our authors and readers, this journal provides supporting information supplied by the authors. Such materials are peer reviewed and may be re-organized for online delivery, but are not copy-edited or typeset. Technical support issues arising from supporting information (other than missing files) should be addressed to the authors.
